# Platelet‐derived transforming growth factor‐β1 promotes keratinocyte proliferation in cutaneous wound healing

**DOI:** 10.1002/term.3022

**Published:** 2020-03-05

**Authors:** Deborah L.W. Chong, Sarah Trinder, Myriam Labelle, Manuel Rodriguez‐Justo, Sian Hughes, Alan M. Holmes, Chris J. Scotton, Joanna C. Porter

**Affiliations:** ^1^ Centre for Inflammation and Tissue Repair UCL, Bloomsbury Campus London UK; ^2^ Department of Inflammation UCL, Royal Free Campus London UK; ^3^ Department of Developmental Neurobiology St. Jude Children's Research Hospital Memphis TN USA; ^4^ Department of Histopathology UCL, Bloomsbury Campus London UK; ^5^ Drug Discovery Group, Translational Research Office, School of Pharmacy UCL London UK; ^6^ Institute of Biomedical and Clinical Sciences University of Exeter Medical School Exeter UK

**Keywords:** blood platelets, epidermis, keratinocytes, platelet‐rich plasma, skin, transforming growth factor beta, transgenic mouse, wound healing

## Abstract

Platelets are a recognised potent source of transforming growth factor‐β1 (TGFβ1), a cytokine known to promote wound healing and regeneration by stimulating dermal fibroblast proliferation and extracellular matrix deposition. Platelet lysate has been advocated as a novel personalised therapeutic to treat persistent wounds, although the precise platelet‐derived growth factors responsible for these beneficial effects have not been fully elucidated. The aim of this study was to investigate the specific role of platelet‐derived TGFβ1 in cutaneous wound healing. Using a transgenic mouse with a targeted deletion of TGFβ1 in megakaryocytes and platelets (TGFβ1^fl/fl^.PF4‐Cre), we show for the first time that platelet‐derived TGFβ1 contributes to epidermal and dermal thickening and cellular turnover after excisional skin wounding. In vitro studies demonstrate that human dermal fibroblasts stimulated with platelet lysate containing high levels of platelet‐derived TGFβ1 did not exhibit enhanced collagen deposition or proliferation, suggesting that platelet‐derived TGFβ1 is not a key promoter of these wound healing processes. Interestingly, human keratinocytes displayed enhanced TGFβ1‐driven proliferation in response to platelet lysate, reminiscent of our in vivo findings. In summary, our novel findings define and emphasise an important role of platelet‐derived TGFβ1 in epidermal remodelling and regeneration processes during cutaneous wound healing.

1

Platelet lysate (PL) or platelet‐rich plasma (PRP) has increasingly been advocated as a novel personalised therapeutic to facilitate tissue repair and regeneration (Anitua, Pino, & Orive, 2017). PL enhances migration and wound closure of keratinocytes or dermal fibroblasts in vitro (Barsotti et al., 2013; Ranzato, Mazzucco, Patrone, & Burlando, 2009). These beneficial effects are presumed to be driven by the high concentration of platelet‐released growth factors such as transforming growth factor‐β1 (TGFβ1) and platelet‐derived growth factor. However, these in vitro studies along with a lack of in vivo data do not address the specific contribution of platelet‐derived TGFβ1 in tissue repair, and in addition, there are no standardised methods of PL or PRP preparation (Marques et al., 2015).

To investigate the role of platelet‐derived TGFβ1 in cutaneous wound healing in vivo, we utilised a targeted knockout transgenic mouse in which megakaryocytes and platelets are depleted of TGFβ1 (TGFβ1^fl/fl^.PF4‐Cre; Labelle, Begum, & Hynes, 2011) in an excisional wound healing model. Normal platelet counts, clotting times, and general physical appearance are reported for these transgenic mice (Labelle et al., 2011). A significant reduction of platelet‐derived active TGFβ1 in transgenic thrombin‐activated PRP compared with littermate controls was confirmed by a TGFβ1 bioassay (Figure 1a). Other growth factors such as PDGF‐BB and VEGF were similar in transgenic thrombin‐activated PRP compared with littermate controls (Figure S1). After 6 mm^2^ punch biopsy excisional wounds were created on the backs of mice, we did not observe any macroscopic differences in the rate of wound closure over 14 days between littermate controls and TGFβ1^fl/fl^.PF4‐Cre animals. This similarity was also reflected in wound width as measured in skin tissue harvested at day 14 after wounding (Figure 1b). This suggests that the loss of platelet‐derived TGFβ1 does not impede the overall healing process. Interestingly, H&E histological analysis of the wound after 14 days revealed significantly thinner epidermis and dermis in TGFβ1^fl/fl^.PF4‐Cre animals compared with controls (Figure 1c,d). Dermal and epidermal layers in unwounded skin surrounding the wound area were similar between control and TGFβ1^fl/fl^.PF4‐Cre mice (Figure 1e). Also, we did not observe any significant difference in the number of hair follicles in the wound site between control and TGFβ1^fl/fl^.PF4‐Cre mice (Figure S2).

**Figure 1:**
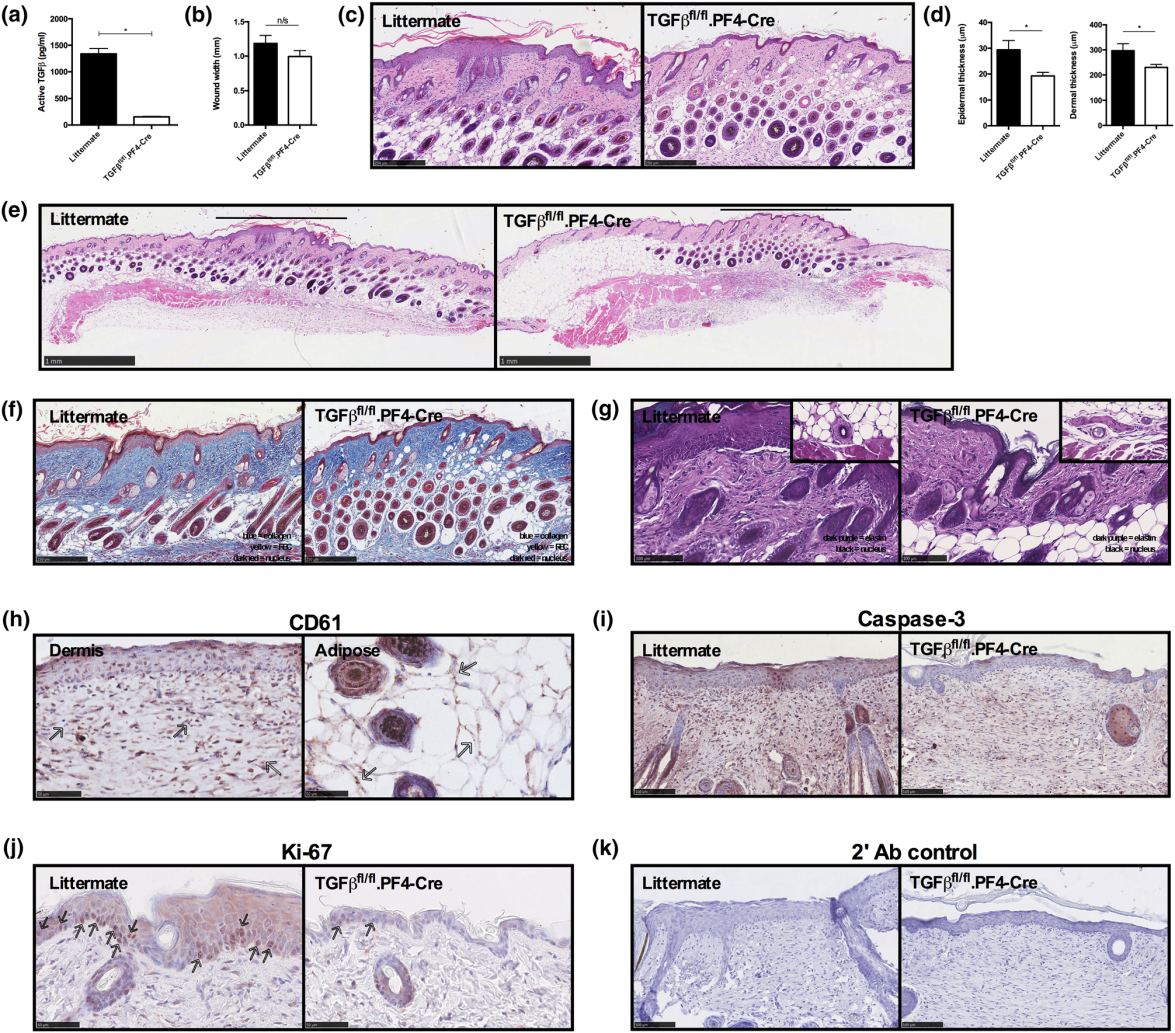
Platelet‐derived transforming growth factor‐β1 (TGFβ1) promotes epidermal hyperplasia in a murine skin wound healing model. (a) Quantification of active TGFβ1 in thrombin activated platelet‐rich plasma from TGFβ1^fl/fl^.PF4‐Cre (*n* = 4) or littermate controls (*n* = 4) by mink lung epithelial cell TGFβ1 bioassay. 6 mm^2^ punch biopsy excisional wounds were made on TGFβ1^fl/fl^.PF4‐Cre or littermate control mice (*n* = 6 per group) and skin tissue harvested 14 days later for histological analysis. (b) Wound width was measured from H&E‐stained skin tissue. (c–d) Representative H&E‐stained wound site at high magnification and quantification of epidermal and dermal thickness from three sites across the wound. (e) Low magnification of H&E‐stained tissue showing surrounding unwounded skin and wound site (marked above by black line). (f) Representative modified Martius scarlet blue trichrome‐stained (blue = collagen, yellow = red blood cell, red = cytoplasm, dark red = nuclei) and (g) elastin‐ and H&E‐stained wounds (dark purple/black = elastin, nuclei = black), insert at higher magnification of blood vessels. (h) CD61, (i) caspase‐3 or (j) Ki‐67 expression, or (k) staining controls as denoted by red staining. CD61^+^ platelets and Ki‐67^+^ cells are indicated by arrows. All image objectives are denoted by the scale bar. Error bars are defined as mean ± standard error of the mean. Any statistical differences using an unpaired *t* test or Mann–Whitney *U* tests are indicated [Colour figure can be viewed at http://wileyonlinelibrary.com]

TGFβ1 is a known mediator of collagen and elastin production from fibroblasts in vitro (Davidson, Zoia, & Liu, 1993). However, modified Martius scarlet blue trichrome and elastin–H&E staining revealed no discernible difference in collagen (blue staining) or elastin deposition (purple staining) at the wound site between control and TGFβ1^fl/fl^.PF4‐Cre mice (Figure 1f,g), although there was a reduction in the elastin staining around the skin capillaries in TGFβ1^fl/fl^.PF4‐Cre mice (Figure 1g insert). This suggests that platelet‐derived TGFβ1 does not significantly regulate extracellular matrix deposition in this model. Furthermore, immunohistochemical analysis showed the presence of CD61^+^ cells (a marker for platelets) throughout the dermal and adipose layers in the wound site (Figure 1h), along with dramatically less caspase‐3 and Ki‐67 expression in TGFβ1^fl/fl^.PF4‐Cre mice compared with staining controls (Figure 1i–k). This indicates that there may be a lower cellular turnover in terms of proliferation and apoptosis in TGFβ1^fl/fl^.PF4‐Cre mice after wounding.

As TGFβ1 has previously been demonstrated to increase over time in wounded skin (Ishida, Kondo, Takayasu, Iwakura, & Mukaida, 2004) and along with our observations of increased in vivo Ki‐67 expression as a marker of cell proliferation, we next investigated whether platelet‐derived TGFβ1 can induce functional changes in specific dermal cells. PL contains high levels of platelet‐derived active and latent TGFβ1 as quantified by a TGFβ1 bioassay (Figure 2a). TGFβ1 treatment of dermal fibroblasts for 48 hr led to significant increase in extracellular collagen I deposition in a concentration‐dependent manner (Figure 2b,c; Figure S3) without affecting cell number compared with media‐treated controls, as quantified by 4′,6‐diamidino‐2‐phenylindole staining. Interestingly, stimulation of dermal fibroblasts with PL did not significantly affect collagen deposition or cell proliferation as determined by EdU incorporation (Figure 2c,d). This suggests that growth factors released from platelets do not have a profound effect on human dermal fibroblast function in vitro. Primary human keratinocytes stimulated with complete media or TGFβ1 had increased cell proliferation, and this was also seen with PL treatment (Figure 2e). TGFβ1‐ or PL‐induced proliferative effects were significantly decreased when keratinocytes were pretreated with an ALK5 inhibitor to block downstream TGFβ1 receptor signalling prior to stimulation with PL (Figure 2e). This suggests that platelet‐derived TGFβ1 significantly contributes to primary human keratinocyte proliferation.

**Figure 2:**
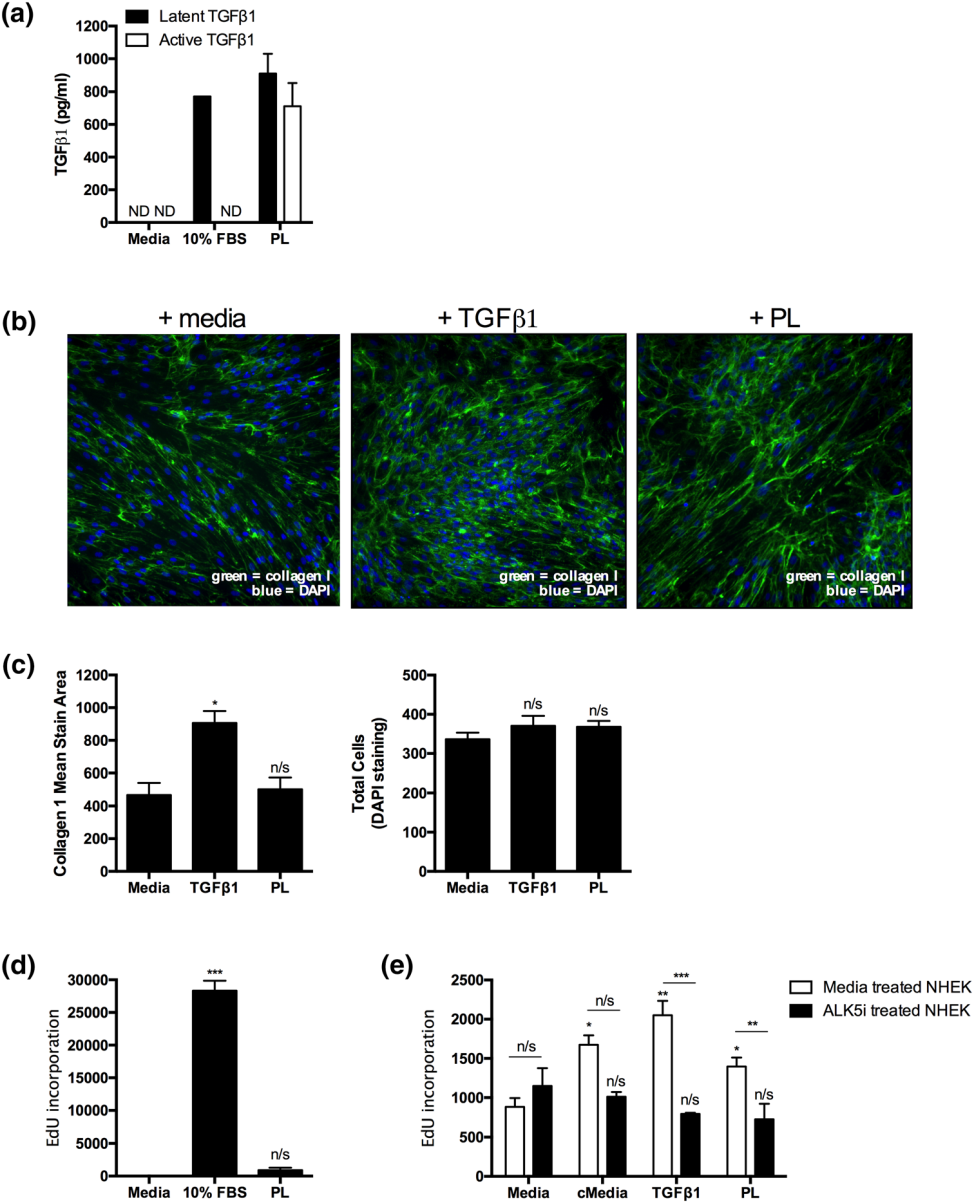
Platelet‐derived transforming growth factor‐β1 (TGFβ1) stimulates epidermal keratinocyte proliferation. (a) Active and latent TGFβ1 quantification of 10% foetal bovine serum (FBS)/Dulbecco's modified eagle's medium (DMEM) or platelet lysate (PL; *n* = 3 donors) by mink lung epithelial cell TGFβ1 bioassay (ND = not detected). (b–c) Representative images and quantification of normal human dermal fibroblast (NHDF; *n* = 2 lines) treated with 1 ng/ml TGFβ1 or 10% PL (*n* = 3 donors) for 48 hr without serum; extracellular collagen I (green) and 4′,6‐diamidino‐2‐phenylindole (blue). NHDF (d) or normal human epidermal keratinocyte (NHEK) (e) treated with 10% PL (*n* = 3 donors), 10% FBS/DMEM, cMedia, or 1 ng/ml TGFβ1 for 72 hr in an EdU Click‐iT assay. NHEKs were pretreated with 1μM ALK5 inhibitor (SB‐525334) for 30 min before stimulation. Proliferation rates as determined by EdU incorporation were calculated relative to treatment‐matched no EdU controls. Error bars are defined as mean ± standard error of the mean. Any statistical differences compared with media control were determined by one‐ or two‐way analysis of variance [Colour figure can be viewed at http://wileyonlinelibrary.com]

As far as we are aware, this is the first study where the specific contribution of platelet‐derived TGFβ1 in an excisional wound healing mouse model has been investigated. Studies investigating the role of TGFβ1 in excisional wound healing models have produced conflicting results. TGFβ1 knockout mice exhibited delayed wound closure with thinner, less vascular granulation tissue (Brown, Ormsby, Doetschman, & Greenhalgh, 1995), whereas mice with keratinocytes overexpressing TGFβ1 display delayed rates of re‐epithelisation after wounding (Chan et al., 2002). We did not witness augmented wound closure or collagen deposition in our platelet‐derived TGFβ1 knockout mice. Perhaps in our model, platelets are not the main source of TGFβ1 in directly mediating wound closure, and other cell types such as keratinocytes may express this important growth factor in vivo to mediate these effects (Amjad, Carachi, & Edward, 2007).

In contrast to other in vitro studies (Ranzato et al., 2009), we did not observe any differences in proliferation or collagen deposition when human dermal fibroblasts were stimulated with PL, even at varying dilutions of PL and time points (Figure S4). This discrepancy may be due to the choice of methodology to measure proliferation (i.e., nuclear crystal violet staining or measuring mitochondrial metabolism compared to EdU incorporation into DNA). Our result of enhanced proliferation of TGFβ1‐stimulated human keratinocytes is novel and differs from other studies (Dahler, Cavanagh, & Saunders, 2001). The discrepancy may reflect differential effects of TGFβ1 dependent on cell‐cycle stage (Alexandrow & Moses, 1995), the amount of TGFβ1 used (10 ng/ml in contrast to our 1 ng/ml), and experimental endpoints (48 vs. our 72 hr). However, TGFβ1 has pleotropic effects and others have shown αv integrin‐activated latent TGFβ1 to increase keratinocyte growth and wound healing in vivo (Duperret, Natale, Monteleon, Dahal, & Ridky, 2016), through ALK‐5‐dependent induction of keratinocyte miRNA‐132 expression, lending support to our in vitro results (Li et al., 2015). Interestingly, TGFβ1 signalling blockade in keratinocytes resulted in decreased proliferative response to PL. These are novel findings as the relative contribution of platelet‐derived TGFβ1 on dermal fibroblasts and keratinocytes has never been specially studied in vitro. These results also mirrored our in vivo mouse data in which we demonstrated increased Ki‐67 staining in control wounded skin, particularly in the epidermal layers, rich in keratinocytes. Less Ki‐67 staining was observed in TGFβ1^fl/fl^.PF4‐Cre animals, suggesting that platelet‐derived TGFβ1 contributes to proliferation of keratinocytes and aids epidermal remodelling.

Of interest, the striking in vivo phenotype observed in our study is reminiscent of aged skin consisting of thinner epidermis (Bhattacharyya, 2004). Although our transgenic animals are not a skin aging model, it has been shown that platelet‐derived mediators such as TGFβ1 are lower in older individuals (Evanson et al., 2014), suggesting that a loss in platelet‐derived TGFβ1 with age may contribute to the histological changes of skin aging.

In conclusion, we have observed a novel role for platelet‐derived TGFβ1 in skin wound healing. Although platelet‐derived TGF1β does not directly enhance the rate of wound closure, it appears to significantly promote keratinocyte proliferation and epidermal layer remodelling and regeneration, and this insightful observation adds to and refines our current understanding of the normal cutaneous wound healing process.

## CONFLICT OF INTEREST

The authors state no conflict of interest.

## AUTHOR CONTRIBUTIONS

ML created the transgenic animals used for this study. DLWC, CJS, AMH, and JCP designed the experiments. DLWC and ST performed the experiments. MRJ and SH analysed the histology images. DLWC analysed the data and wrote the manuscript. All authors edited the manuscript.

## Supporting information


**Figure S1** Quantification of PDGF‐BB and VEGF in thrombin activated PRP from TGFβ1^fl/fl^.PF4‐Cre (*n* = 4) or littermate controls (*n* = 4) by ELISA. Error bars are defined as mean ± SEM. No significant differences were found using a Mann Whitney U test.
**Figure S2** 6mm^2^ punch biopsy excisional wounds were made on TGFβ1^fl/fl^.PF4‐Cre or littermate control mice (*n* = 6/group) and skin tissue harvested 14 days later for histological analysis. The number of hair follicles in the wound site were counted on H&E stained sections. Error bars are defined as mean ± SEM. No significant different was found using a student t‐test.
**Figure S3** NHDFs were treated with varying TGFβ1 concentrations for 48 h and extracellular collagen deposition and total cell number were quantified by immunofluorescent staining. Error bars are defined as mean ± SEM. Any significant differences were determined by 1‐way ANOVA compared to the media control and indicated above.
**Figure S4** NHDFs were treated with varying PL amounts, 10% FBS (positive control) or media (negative control) for 24, 48 or 72 h. Cell proliferation were quantified by an EdU incorporation Click‐iT assay. Error bars are defined as mean ± SEM. Any significant differences were determined by 1‐way ANOVA compared to the time‐matched media control and indicated above.
