# Supplementary material for: Platelet‐derived transforming growth factor‐β1 promotes keratinocyte proliferation in cutaneous wound healing
Source: J Tissue Eng Regen Med. 2020 Mar 5;14(4):645–9. doi: 10.1002/term.3022 (PMC7216944; doi:10.1002/term.3022)
Supplement: Supplementary file 1 — Figure S1 Quantification of PDGF‐BB and VEGF in thrombin activated PRP from TGFβ1fl/fl.PF4‐Cre (n = 4) or littermate controls (n = 4) by ELISA. Error bars are defined as mean ± SEM. No significant differences were found using a Mann Whitney U test. Figure S2 6mm2 punch biopsy excisional wounds were made on TGFβ1fl/fl.PF4‐Cre or littermate control mice (n = 6/group) and skin tissue harvested 14 days later for histological analysis. The number of hair follicles in the wound site were counted on H&E stained sections. Error bars are defined as mean ± SEM. No significant different was found using a student t‐test. Figure S3 NHDFs were treated with varying TGFβ1 concentrations for 48 h and extracellular collagen deposition and total cell number were quantified by immunofluorescent staining. Error bars are defined as mean ± SEM. Any significant differences were determined by 1‐way ANOVA compared to the media control and indicated above. Figure S4 NHDFs were treated with varying PL amounts, 10% FBS (positive control) or media (negative control) for 24, 48 or 72 h. Cell proliferation were quantified by an EdU incorporation Click‐iT assay. Error bars are defined as mean ± SEM. Any significant differences were determined by 1‐way ANOVA compared to the time‐matched media control and indicated above. [file TERM-14-645-s001.docx]

**Supporting information**

**Human platelet lysate samples, primary human dermal cells and mice**

Human whole blood from healthy controls was collected with written consent and research ethics committee approval. Platelet lysates (PL) were obtained by centrifuging whole blood collected in EDTA tubes for 8 min at 800 *g*. The resultant platelet-rich plasma top fraction was freeze/thawed three times before centrifugation to remove the cell debris to yield PL. Primary human dermal fibroblasts (NHDF) and keratinocytes (NHEK) were purchased from Promocell UK. NHDF were maintained in 10% FBS/DMEM (Invitrogen, UK) at 10% CO_2_ and NHEK were maintained in complete Keratinocyte Growth Medium 2 supplemented with BPE, hEGF, hydrocortisone, insulin, epinephrine, transferrin and CaCl_2_ (designated cMedia; PromoCell, UK) at 5% CO_2_ in mycoplasma-free conditions and used by passage 5 from receipt. TGFβ1^fl/fl^.PF4-Cre mice were obtained from Prof Richard Hynes (Labelle et al., 2011) and all animals were housed and maintained with accordance to UK Home Office legislation under the Animals (Scientific Procedures) Act 1986 Amendment Regulations (SI 2012/3039).

**Skin wound healing model**

Two 6mm^2^ full-thickness excisional wounds were made either side of the midline under general anaesthesia on clean shaven backs of 11-18 weeks old TGFβ1^fl/fl^.PF4-Cre or littermate controls. The wound diameter was routinely measured before animals were euthanised at day 14 post wounding and tissue harvested for histology and immunohistochemistry (IHC).

**Histology & IHC**

Murine skin samples were embedded in paraffin wax blocks. 5μm sections were stained with Hematoxylin & Eosin (H&E) or modified Martius Scarlet Blue (MSB) Trichrome staining protocol using an automated slide stainer (Sakura Tissue-Tek DRS 2000, Japan). Elastin-H&E staining using Miller’s elastin solution prior to counterstaining with Harris haematoxylin was performed manually. IHC was performed for the presence of murine CD61 (clone AB-7773, Sigma-Aldrich, UK), Ki-67 (clone ab-15580, Abcam, UK), Caspase-3 (clone ab13847, Abcam, UK) or human CD61 (clone 2f2, Leica Biosystems, Germany) under standard heat-induced epitope retrieval methods with citrate buffer pH 6.0. An additional blocking step with 4% goat serum/1% BSA/PBS was performed prior to incubation with the Ki-67 primary antibody. Staining was developed using HRP-conjugated secondary antibody and NovaRED Peroxide (HRP) substrate kit (all from Vector Laboratories, UK) and counter-stained with Haematoxylin. All sections were scanned on a Nanozoomer Digital Slide Scanner and images analysed using NDP.view software (both from Hamamatsu Corporation, Japan).

***In vitro* culture of NHDF or NHEK with PL**

Extracellular collagen deposition from stimulated primary dermal fibroblasts (NHDF) was measured in a molecular crowding assay (Chen et al., 2009). In brief, serum-starved NHDF were stimulated with 1 ng/ml (unless stated otherwise) TGFβ1, (R&D systems, UK) or 10% PL for 48h before fixation in ice-cold methanol. Samples were then stained with anti-collagen I primary antibody (clone Col-1, Sigma-Aldrich, UK) overnight at 4°C before detection with a AF488-conjugated secondary anti-mouse antibody and nuclear counter-stained with DAPI (both from Invitrogen, UK). Staining was quantified on an ImageXpress Micro XLS Widefield High-Content Analysis System (Molecular Devices, USA). Rates of proliferation were determined by an EdU incorporation click-iT proliferation assay (Invitrogen, UK). In brief, NHDF cultured in DMEM or primary human keratinocytes (NHEK) cultured in basal media (without BPE and hEGF) were stimulated with 10% FBS, 10% (unless stated otherwise) PL, 1 ng/ml rTGFβ1 or cMedia and 10 μM EdU spiked into wells in the last 16h of a 72h (unless stated otherwise) incubation period. Fluorescent readings were taken after performing the click-iT reaction following manufacturer’s protocols and the rate of proliferation was calculated relative to no EdU controls. For ALK5 inhibition studies, NHEK were pre-treated with 1μM SB-525334 (Sigma-Aldrich, UK) 30 min prior to stimulation.

**Quantification of Growth Factors**

Levels of active TGFβ1 were measured in media, human PL or murine PRP pre-activated with 0.5 U/ml thrombin for 20min by Mink Lung Epithelial Cell (MLEC) TGFβ1 bioassay. In brief, MLEC stably transfected with an expression construct containing the plasminogen activator inhibitor-1 (PAI-1) promoter fused to a firefly luciferase reporter gene were treated with samples for 14 hours. Bioluminescence was measured after the addition of luciferin substrate (Promega, UK). PDGF and VEGF in murine PRP pre-activated with thrombin were quantified by commercial available ELISA kits (R&D systems and Generon, UK respectively).

**Statistical analysis**

Statistical analysis was performed with Prism 6 (GraphPad software, USA) using either unpaired student t-test, Mann Whitney U test, 1- or 2-way ANOVA with Holm-Sidak post-hoc testing. Mean ± SEM are shown in all graphs with any statistical significances indicated (*p<0.05, **p<0.01, ***p<0.001).

**Supplemental Figure 1**


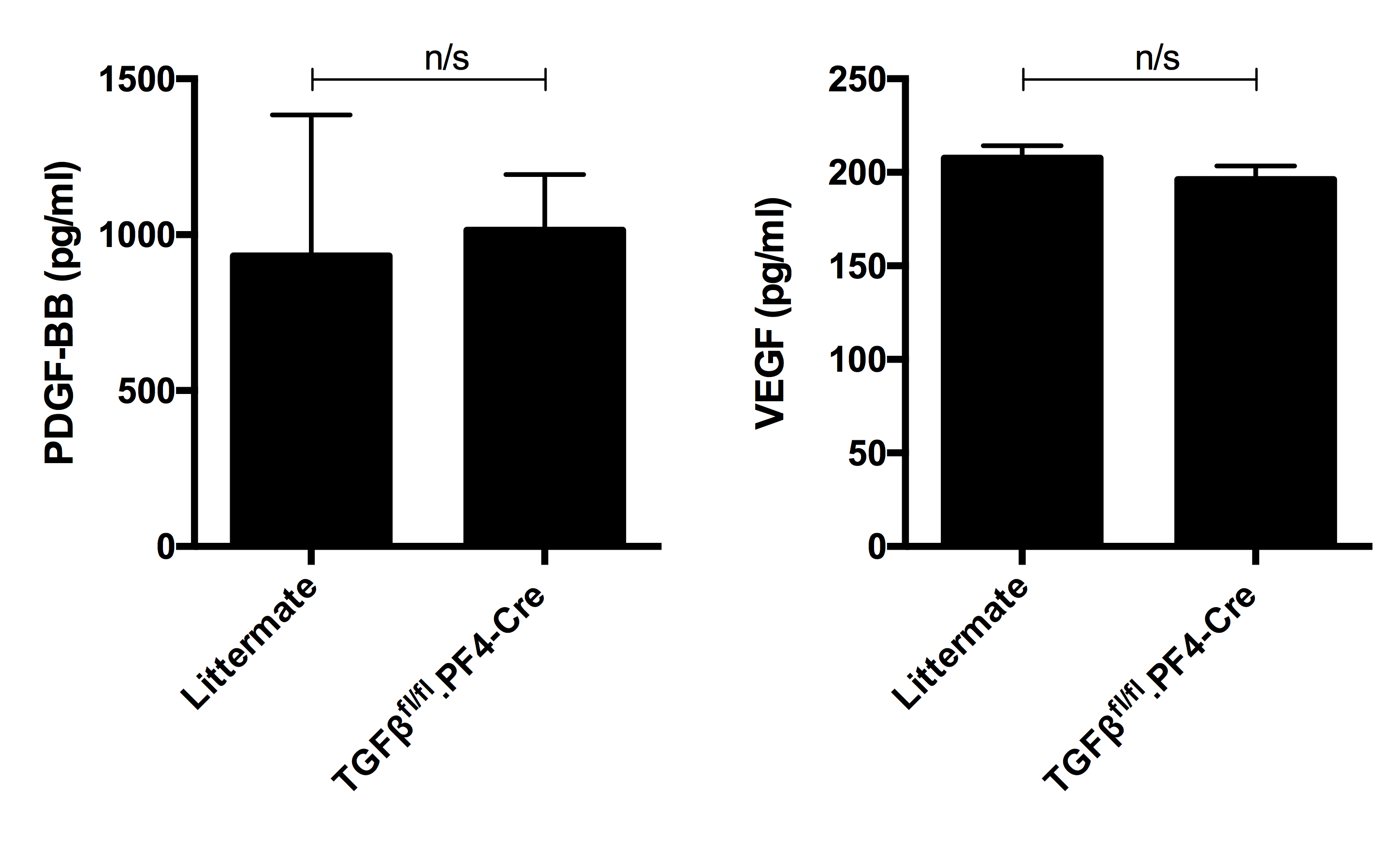


Quantification of PDGF and VEGF in thrombin activated PRP from TGFβ1^fl/fl^.PF4-Cre (*n*=4) or littermate controls (*n*=4) by ELISA. Error bars are defined as mean ± SEM. No significant differences were found using a Mann Whitney U test.

**Supplemental Figure 2**


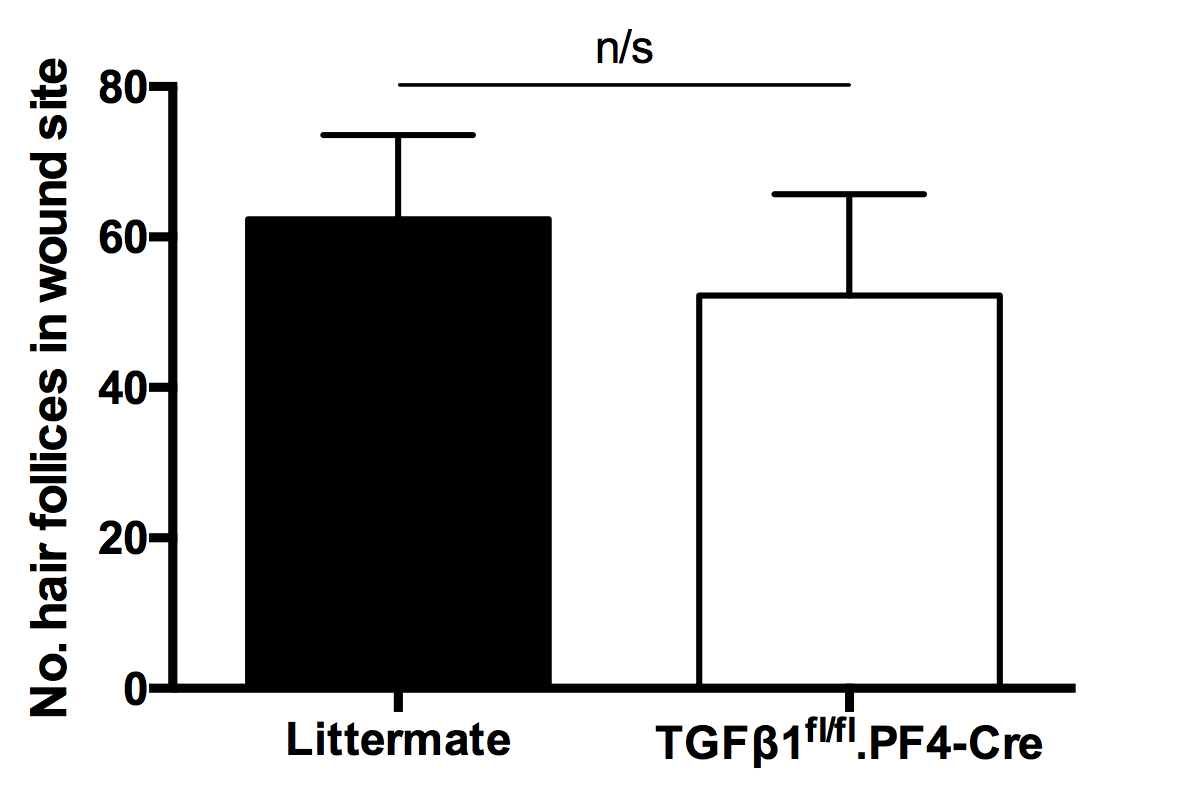


6mm^2^ punch biopsy excisional wounds were made on TGFβ1^fl/fl^.PF4-Cre or littermate control mice (*n*=6/group) and skin tissue harvested 14 days later for histological analysis. The number of hair follicles in the wound site were counted on H&E stained sections. Error bars are defined as mean ± SEM. No significant different was found using a student t-test.

**Supplemental Figure 3**


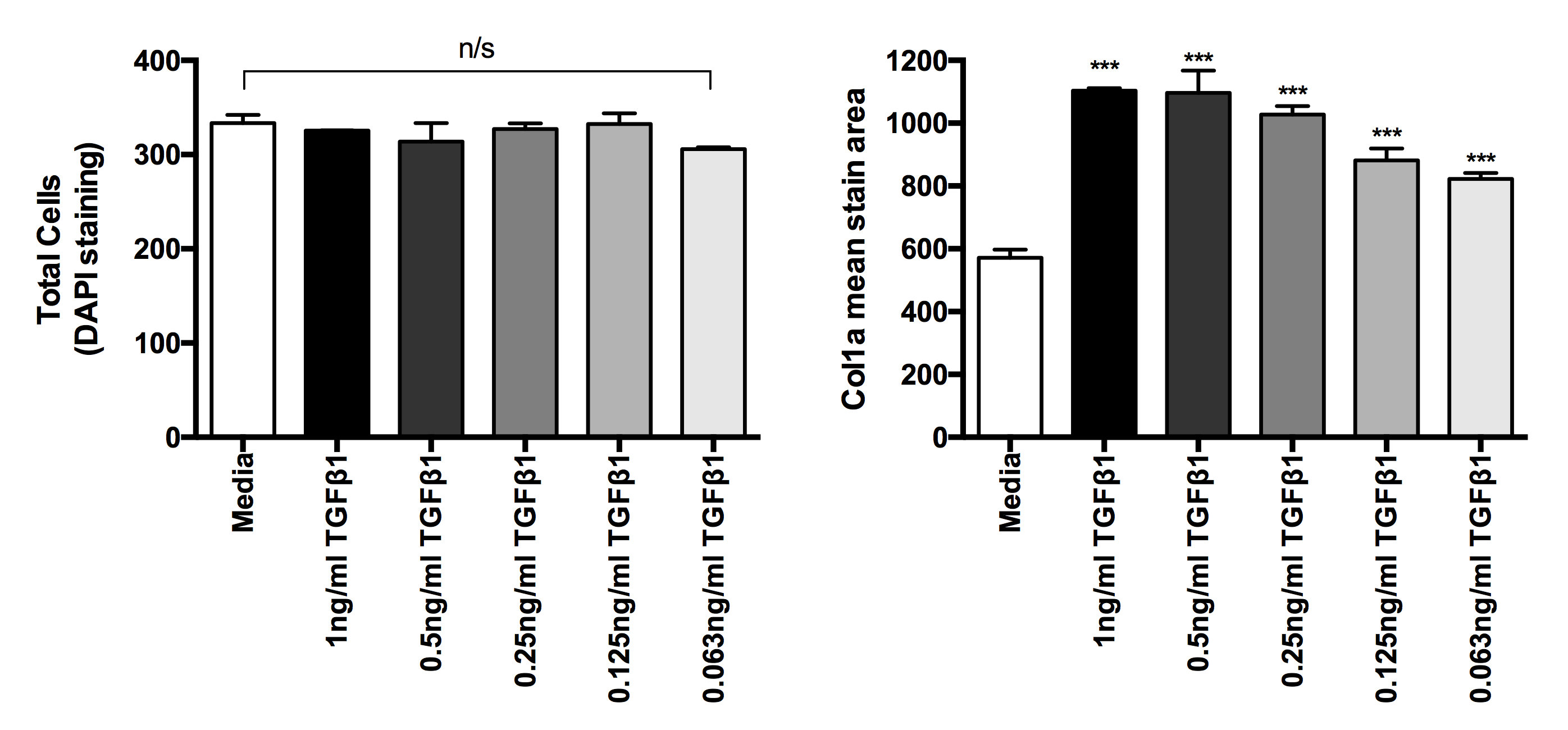


NHDFs were treated with varying TGFβ1 concentrations for 48h and extracellular collagen deposition and total cell number were quantified by immunofluorescent staining. Error bars are defined as mean ± SEM. Any significant differences were determined by 1-way ANOVA compared to the media control and indicated above.

**Supplemental Figure 4**


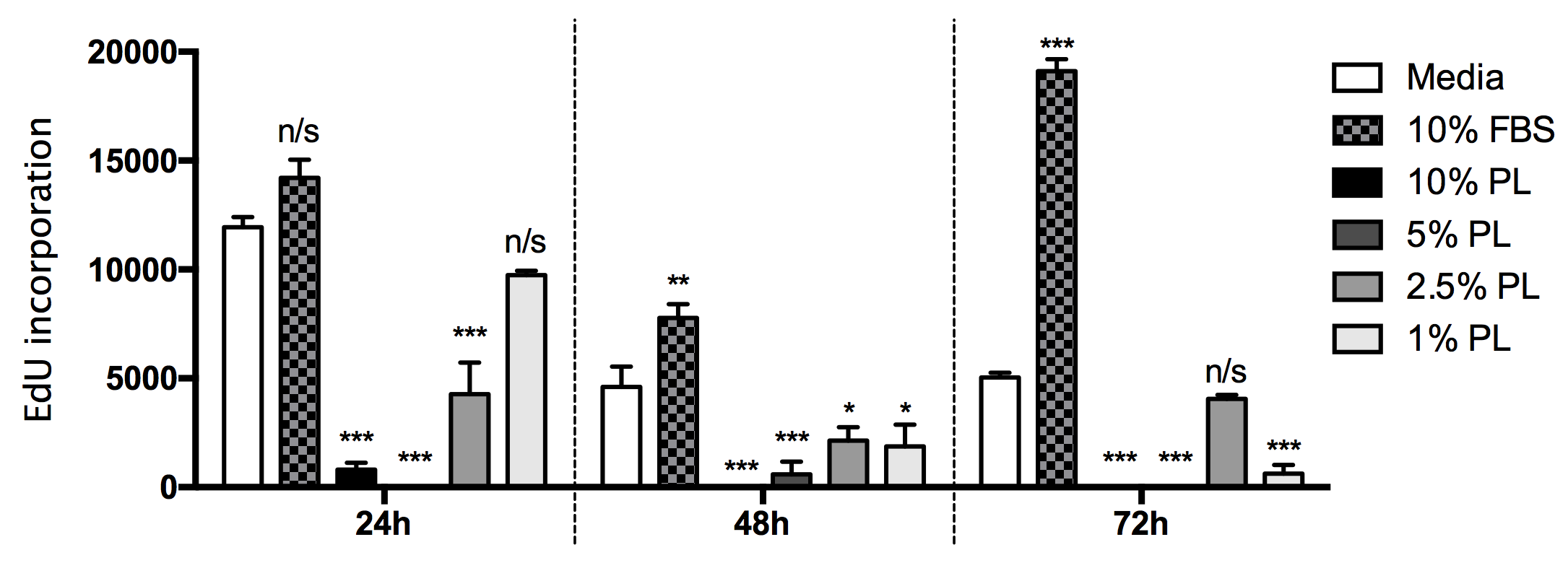


NHDFs were treated with varying PL amounts, 10% FBS (positive control) or media (negative control) for 24, 48 or 72h. Cell proliferation were quantified by an EdU incorporation Click-iT assay. Error bars are defined as mean ± SEM. Any significant differences were determined by 1-way ANOVA compared to the time-matched media control and indicated above.
